# The characterization of conserved binding motifs and potential target genes for *M. tuberculosis *MtrAB reveals a link between the two-component system and the drug resistance of *M. smegmatis*

**DOI:** 10.1186/1471-2180-10-242

**Published:** 2010-09-16

**Authors:** Yuqing Li, Jumei Zeng, Hua Zhang, Zheng-Guo He

**Affiliations:** 1 National Key Laboratory of Agricultural Microbiology, Center for Proteomics Research, College of Life Science and Technology, Huazhong Agricultural University, Wuhan 430070, China

## Abstract

**Background:**

The two-component systems of *Mycobacterium tuberculosis *are apparently required for its growth and resistance in hostile host environments. In such environments, MtrAB has been reported to regulate the expression of the *M. tuberculosis *replication initiator gene, *dnaA*. However, the *dnaA *promoter binding sites and many potential target genes for MtrA have yet to be precisely characterized.

**Results:**

In this study, a 7 bp sequence motif in the *dnaA *promoter region was identified for MtrA binding using DNaseI footprinting assays and surface plasmon resonance (SPR) analysis. Approximately 420 target genes potentially regulated by MtrA, including the isoniazid inducible gene *iniB*, were further characterized from *M. tuberculosis *and *M. smegmatis *genomes. When assayed using quantitative real-time PCR (qRT-PCR), many of the target genes demonstrated significant expression changes when the antisense mRNA of the *mtrA *gene was expressed in *M. smegmatis*. The recombinant mycobacteria grew in length and were more sensitive to two anti-tuberculosis drugs, isoniazid and streptomycin.

**Conclusions:**

These findings yield critical information about the regulatory mechanisms of the MtrAB two-component system and its role in the drug resistance of *M. smegmatis*.

## Background

*M. tuberculosis *is one of the most devastating human pathogens, and its threat to human health has intensified with the emergence of multidrug-resistant tuberculosis (TB) and the worldwide prevalence of co-infection with HIV [1,2]. Two-component regulatory systems (TCRs) are widely distributed among bacteria and plants and enable organisms to regulate gene expression in response to a variety of environmental stimuli [3,4]. Some TCRs are clearly involved in regulating the virulence of pathogenic bacteria [3].

The *M. tuberculosis *genome contains 11 paired TCRs and several orphan kinases and regulators [5]. Several TCRs are apparently required for the growth of *M. tuberculosis *under specific conditions [6-8]; for example, *mprA-mprB *is important for the maintenance of persistence [6]. Of the 11 *M. tuberculosis *TCRs, only the *mtrA-mtrB *system has been confirmed as essential for *in vitro *and *in vivo *survival of *M. tuberculosis *[9-11]. Several recent reports show that the regulator MtrA modulates *M. tuberculosis *proliferation by regulating *dnaA *expression and binding the origin of replication [12,13]. In *Mycobacterium avium*, morphotypic multidrug resistance requires the presence of an MtrA homologue [14].

The *mtrAB *system has been successfully deleted in *Corynebacterium glutamicum*, an industrial amino acid production strain [15]. Mutant cells lacking *mtrAB *showed a different cell morphology and were more sensitive to penicillin, vancomycin, and lysozyme, however, they were more resistant to ethambutol [15]. The expression of some genes involved in both peptidoglycan metabolism and osmoprotection was also substantially changed [15]. Therefore, MtrAB in *C. glutamicum *is thought to be involved in regulating cell wall metabolism and osmoprotection.

The *M. tuberculosis *MtrAB system is thought to be involved in the expression of many target genes and contributes to the pathogen survival and resistance within its host tissue. However, these target genes and their MtrA binding sites have not been clearly established. In the current study, we have identified conserved sites for the recognition of MtrA in the *dnaA *promoter, as well as approximately 420 potential target genes. Further *in vivo *studies concerning a related organism, *M. smegmatis*, reveal changes in both cell morphology and drug resistance when MtrA gene expression is inhibited. The data presented here significantly enhance our understanding of the regulatory mechanisms of the essential two-component MtrAB system and its role in mycobacterial drug resistance.

## Results

### MtrA interacted with the regulatory region of the *M. tuberculosis dnaA *gene

**Figure 1:**
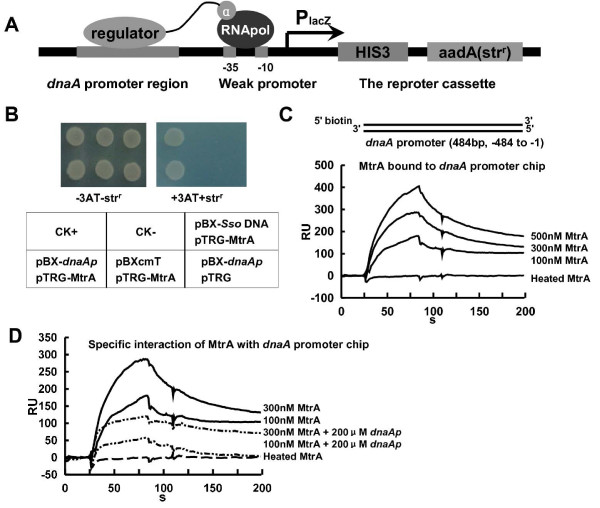
**Two-component regulator MtrA interacts with the regulatory region of *dnaA***. **(A) **The regulatory sequence of the *dnaA *initiator gene was cloned into the reporter genes upstream of *HIS3-aadA *of the reporter vector pBXcmT (24). **(B) **The interaction between MtrA and the promoter region of *dnaA *was measured by bacterial one-hybrid analysis. Upper panel: bacterial two-hybrid plates. Lower panel: an outline of the plates in the upper panel. Each unit represents the corresponding co-transformant in the plates. CK^+^: co-transformant containing pBX-Rv2031p and pTRG-Rv3133c as a positive control (24). CK^-^: co-transformant containing pBX-Rv2031p and pTRG-Rv3133c-deltaC as a negative control (24). SsoDNA, an unrelated archaeal DNA sequence, was also used a negative control. **(C) **SPR assays for the binding of *dnaA *promoter chip by MtrA. **(D) **The specific interaction between the regulatory region of the *M. tuberculosis dnaA *gene was assayed by SPR. Unlabeled promoter DNA was used as competition for the binding of MtrA with DNA on chip. An overlay plot was produced to show the interactions.

Bacterial one-hybrid assays confirmed the interaction between MtrA and the regulatory sequence of the *dnaA *initiator gene. The *dnaA *promoter region was cloned into the reporter genes upstream of *HIS3-aadA *and the reporter vector pBXcmT (Fig. 1A). As shown in Fig. 1B, the co-transformant strain with the *dnaA *promoter and MtrA was observed to grow well on the screening medium. In contrast, there was no growth for the strain containing either MtrA or the *dnaA *promoter alone. In addition, neither the co-transformant strain containing an unrelated DNA, SsoDNA (Additional file 1), nor MtrA did grew, indicating that this DNA cannot interact with MtrA (Fig. 1B). Thus, MtrA specifically interacted with the *dnaA *gene promoter.

The interaction of the purified MtrA protein with the *dnaA *promoter was confirmed by the interaction with the DNA on the chip. As shown in Fig. 1C, the biotinylated promoter DNA was first associated with the streptavidin (SA) chip (GE Healthcare). When an increasing concentration of MtrA protein (100-500 nM) was passed over the chip surface, a corresponding increasing response value was observed. This again indicated that the MtrA protein could bind with the *dnaA *promoter DNA (Fig. 1C). In contrast, heated inactive protein showed no response when it was passed over the chip (Fig. 1C). When an unspecific DNA, the promoter of Rv0467, was coated on the chip, no significant association for MtrA was observed (Additional file 2). In a further confirmatory experiment, 200 μM unlabeled promoter DNA was also added along with the MtrA protein. This DNA competed with that on the chip for the available MtrA; here, a significantly lower response was observed compared to a control with no competition (Fig. 1D).

### Characterization of the DNA-box motif in the dnaA promoter that allows MtrA binding

**Figure 2:**
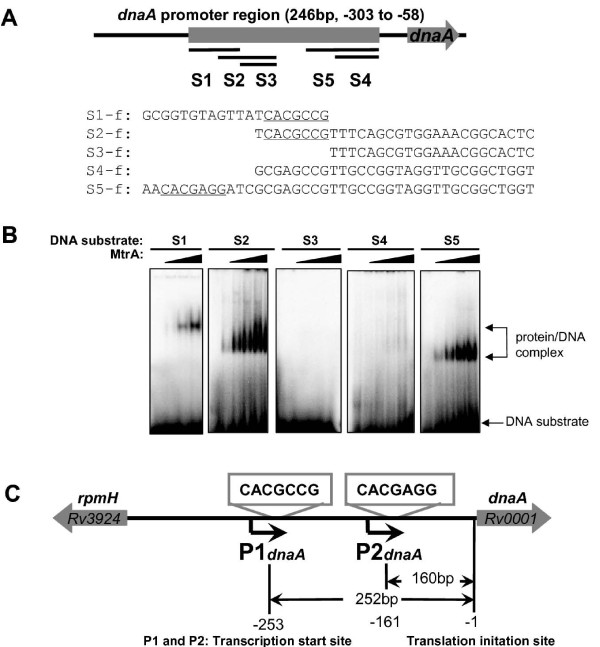
**Characterization of the sequence motifs for MtrA in the *M. tuberculosis dnaA *gene promoter region**. The DNA-binding assays of *M. tuberculosis *MtrA were performed using modified EMSA and SPR assays, as described in "Materials and Methods". **(A) **Several short DNA fragments were synthesized and used as DNA substrates, which covered a different *dnaA *gene promoter region. The sequences of their positive strands are indicated, and two 7 bp motifs are underlined. **(B) **The DNA-binding assays for MtrA on different DNA substrates. The EMSA reactions (10 μl) for measuring the mobility shift contained 200 fmol ^32^P-labeled DNA and increasing amounts of MtrA proteins (100 nM-600 nM). The protein/DNA complex is indicated by arrows on the right of the panels. **(C) **Schematic representation of conserved motifs located downstream of two *dnaA *promoters. The base-pair numbers far from the start codon of the *dnaA *gene are indicated.

Several short DNA fragments (S1-S5) were used to precisely determine the DNA-box motif for the MtrA in this promoter region (Fig. 2A). As shown in Fig. 2B, a specific protein/DNA complex was observed on S1, S2, and S5, indicating that MtrA could recognize these DNA substrates. In contrast, no binding activity was observed for substrates S3 and S4, both of which lacked the 5-CACGCCG-3 or 5-CACGAGG-3 sequence box (Fig. 2A). Further confirmation of the specific interaction was obtained by conducting the competing surface plasmon resonance (SPR) assay with the unlabeled DNA fragments. As shown in Additional file 3, a significantly lower response was observed when either the unlabeled S2 or S5 was added together with MtrA, which indicated that they could compete the binding of MtrA with the promoter DNA on the chip. Therefore, these two sequence motifs appeared to be essential for the MtrA binding with the *dnaA *regulatory region.

**Figure 3:**
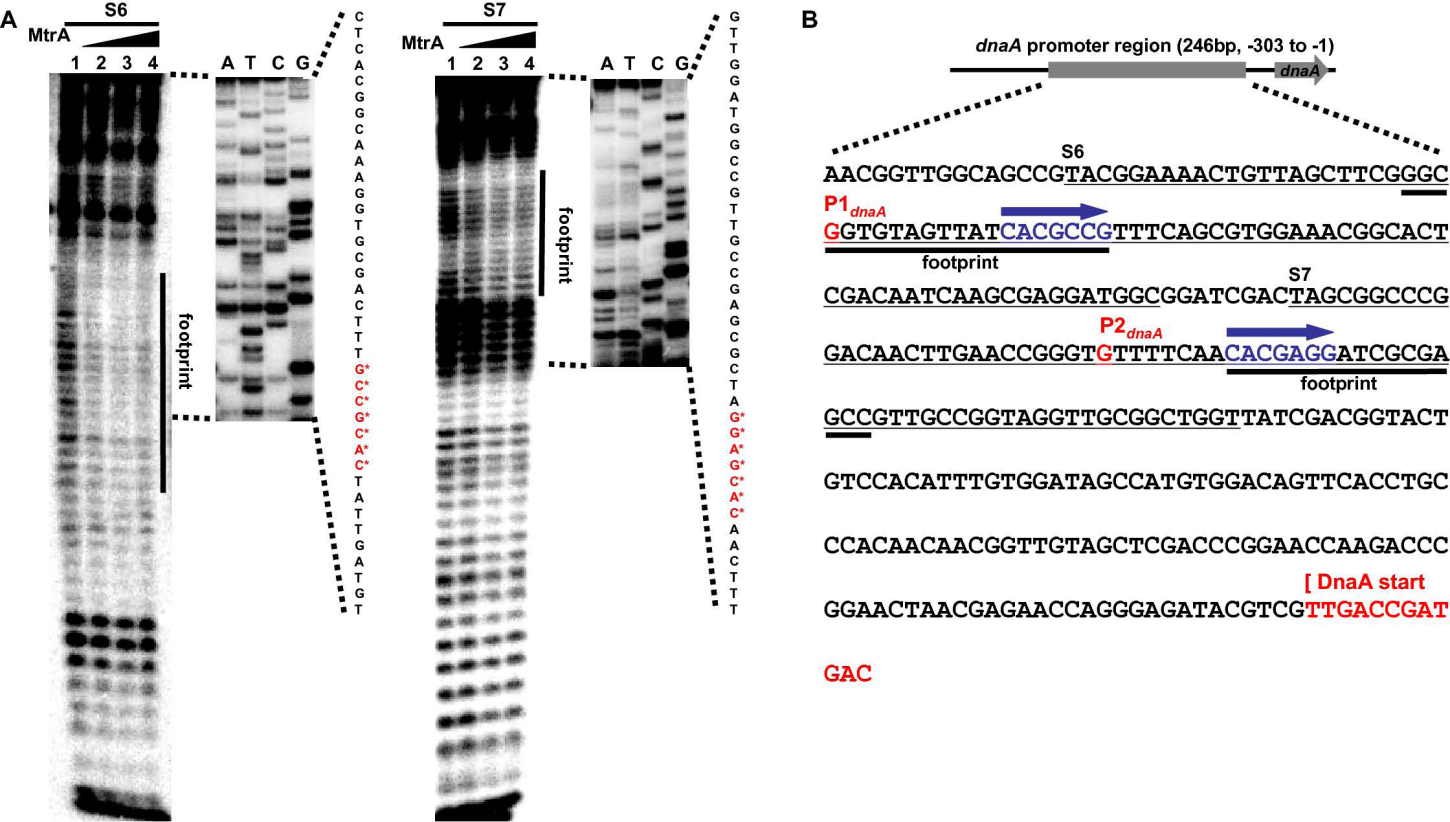
**MtrA footprinting analysis in the *M. tuberculosis dnaA *promoter region**. **(A) **DNase I footprinting assay of the protection of two short *dnaA *promoter regions (S6 and S7) against DNase I digestion by MtrA. The substrate S6 contains S1 and S2 sequences, and the substrate S7 contains S5 sequences. The ladders are shown in the right panel and the obtained nucleotide sequences are listed. The protected regions are indicated. The two specific sequence boxes are indicated by "*". **(B) **Summary of MtrA footprinting analysis in the *M. tuberculosis dnaA *promoter region. The DNA sequence correspond with the *dnaA *promoter region from -303 to -1. The position of two transcription start sites (P1_dnaA _and P2_dnaA_), two footprint regions, and two MtrA binding boxes are indicated.

The interaction between MtrA and these two sequence boxes was further confirmed by DNase I footprinting assays (Fig. 3). Regions that contain these two boxes were significantly protected when MtrA was present. Protection at S6 occurred at all MtrA concentrations while the protection of S7 was dependent on the concentration of MtrA. This suggests that MtrA has different binding affinities with these regions.

We characterized two sequence boxes for the recognition of MtrA within the *dnaA *promoter, situated immediately downstream of promoters P1 and P2. The binding sequence boxes and their situation within the *dnaA *promoter are summarized in Fig. 2C.

### Characterization of potential target genes regulated by MtrA in mycobacterial genomes

**Figure 4:**
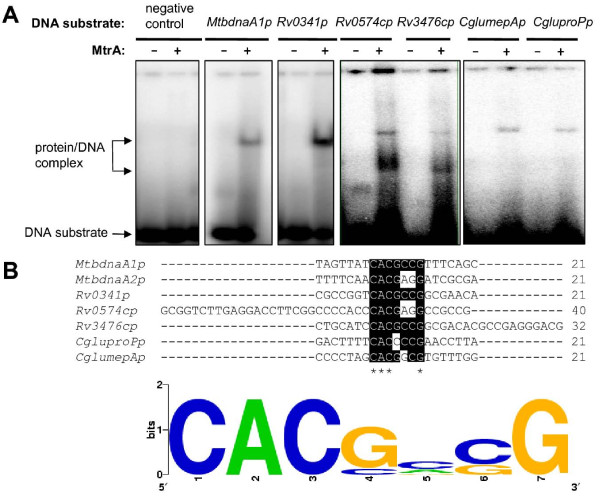
**Characterization of the conserved sequence motif for MtrA in mycobacteria and *C. glutamicum***. **(A) **EMSA assays for validating the binding of MtrA with regulatory sequences of several potential target genes from *M. tuberculosis*. The promoter DNA of *M. tuberculosis dnaA *gene was used as positive control. An unrelated DNA was used as negative control. Several DNA substrates, namely, *Rv0341_up*, *Rv0574c_up*, and *Rv3476c_up*, were amplified from their promoter regions using specific primers. Several regulatory sequences of potential target genes from *C. glutamicum *including *CglumepAp *and *CgluproPp*, were amplified and used as DNA substrates. **(B) **A blast assay for the conserved sequence motif recognition by MtrA. Sequence alignment was carried and visualized by local BioEdit software. The complete consensus sequence is indicated by the stars under the base in the upper panel. Sequence logo was generated by WebLogo tool.

We searched the intergenic regions of the *M. tuberculosis *and *M. smegmatis *genomes extensively based on the two sequence motifs for MtrA in the *dnaA *gene promoter region. To validate the target genes, several regulatory regions of the genes were amplified. The DNA-binding activities of MtrA were examined using EMSA assays. As shown in Fig. 4, the regulatory sequence of a predicted target gene, isoniazid inducible gene *iniB *(*rv0341*), could be recognized by MtrA. A specific DNA/protein complex band was also observed. In addition, MtrA was able to bind with two target promoter DNA sequences of Rv0574 (a hypothetical protein) and Rv3476 (KgtP), producing a corresponding DNA/protein band (Fig. 4A). The positive target DNA was shown to bind with MtrA, while the negative DNA was not. The 7 bp sequence motif could also be found in the promoter regions of two previously characterized target genes, *CgmepA *and *CgproP*, in *C. glutamicum*. Interestingly, *M. tuberculosis *MtrA bound with the regulatory sequences and produced specific shifted bands (Fig. 4A).

A further logo assay for the consensus sequence was conducted using the WebLogo tool [16]. A more general conserved motif for MtrA recognition was mapped out (Fig. 4B). In all, 155 potential target genes were characterized from the *M. tuberculosis *genome (Additional file 4), and 264 genes were characterized from the *M. smegmatis *genome (Additional file 5).

### Effects of mtrA gene expression level on mycobacterial drug resistance and cell morphology

**Figure 5:**
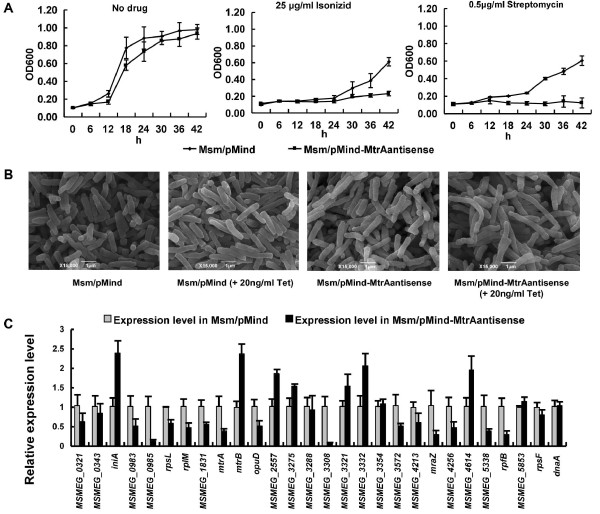
**Effects of the expression level of *mtrA *gene on target genes and cell growth in *M. smegmatis***. **(A) **Drug resistance assays. The antimicrobial activity of four first-line anti-tuberculosis drugs against *M. smegmatis *was determined as described under "Materials and Methods". Representative growth curves for isonizid and streptomycin are shown. **(B) **Scanning electron microscopy assay of cell morphology. The experiment was carried out as described in the "Materials and Methods". Representative images are shown. The images were taken at 15,000× magnification. Bars, 1 μm. **(C) **qRT-PCR assays for the gene expression of *M. smegmatis*. The experiment was carried out as described in the "Materials and Methods". 16S rRNA gene, *rrs*, was used as control. All target genes were amplified using specific primers. Different gene expressions were normalized to the levels of 16S rRNA gene transcripts, and the folds of expression change were calculated. Representative data are shown.

The mRNA antisense expression of the *mtrA *gene in *M. smegmatis *showed a regulatory effect of *mtrA *on mycobacterial drug resistance and cell morphology [17]. No substantial change was observed for the general growth of the recombinant mycobacterial strains. However, as shown in Fig. 5A, the recombinant mycobacterial cells became sensitive to the anti-TB drugs isoniazid and streptomycin, as evidenced by their inhibited growth in the presence of 25 μg/mL of isoniazid or 0.5 μg/mL of streptomycin in the medium. In contrast, no noticeable inhibition was observed for two other drugs, ethambutol and rifampicinB (data not shown). With a general growth of the recombinant mycobacterial strains resulting in minimal change, the cell morphology was further examined using the scanning electron microscopy (SEM) technique. As shown in Fig. 5B, the cell lengthened when 20 ng/mL tetracycline was added to the medium to induce expression of the antisense *mtrA *mRNA (right panel).

When relative gene expression was measured via qRT-PCR as shown in Fig. 5C, the *mtrA *gene was only 0.38-fold that of the wild-type strain, indicating that the expression of the *mtrA *gene in recombinant *M. smegmatis *was greatly inhibited. The expression of the *dnaA *gene in the recombinant strain basically remained constant when compared with that in the wild-type strain. This was consistent with the fact that no conserved sequence motif existed within the regulatory region of this gene in *M. smegmatis*. Another approximately 26 potential target genes were randomly chosen to measure the expression change in the recombinant *M. smegmatis *strain (Fig. 5C). The expression levels of these genes clearly changed; *iniA *and *mtrB *gene expression increased 2.5-fold expression (Fig. 5C), while *mraZ *(Msmeg_4236) and *rpfB *(Msmeg_5439) gene expression decreased by about 0.2-fold (Fig. 5C).

Therefore, the inhibition of the *mtrA *gene resulted in corresponding expression changes in many predicted target genes in *M. smegmatis*. The expression level of the *mtrA *gene consequently affected the drug resistance and cell morphology of *M. smegmatis*.

## Discussion

MtrAB has been reported to regulate the expression of the *M. tuberculosis *replication initiator gene, *dnaA *[12]. However, potential binding sites for MtrA have not been clearly characterized. In addition, there are many potential target genes that also appear to be regulated by MtrA. In the current study, we identified a 7 bp conserved sequence motif for the recognition of MtrA within the *dnaA *promoter. About 420 potential target genes regulated by MtrAB were predicted from the *M. tuberculosis *and *M. smegmatis *genomes upon searching their promoter databases. Many predicted target genes showed significant expression changes when the *mtrA *homologue of *M. smegmatis *was partially inhibited. The recombinant *M. smegmatis *cells increased in length and became sensitive to the anti-TB drugs isoniazid and streptomycin.

The transcription of *dnaA *starts essentially at P1*_dnaA_*, which is conserved in all mycobacterial species [18]. The analysis of the sequence in the upstream region of *dnaA *revealed a second promoter, P2*_dnaA, _*in *M. tuberculosis *[18]. In previous *in vivo *experiments, MtrA bound with the regulatory region of the *dnaA *gene [12]. In the current study, two binding motifs for MtrA were located immediately downstream from the two promoters (Fig. 2C). Therefore, MtrA can apparently interfere with the promoter activity and thus regulate the expression of the replication initiator gene. The promoter P2*_dnaA _*only exists in a viral strain or derivative strains such as *M. tuberculosis*, *M. bovis *and BCG, but not in *M. avium *or *M. smegmatis *[18]. This suggests that the two-component system MtrAB might contribute to the virulence of the *M. tuberculosis *complex through selective regulation of *dnaA *gene expression. A parallel study [13] has identified a "GTCACAgcg" motif for the recognition of MtrA in the *fbpB *promoter and the origin of replication. Interestingly, there exists a common conserved core sequence between the 9 bp motif and the motif identified within the *dnaA *promoter in the current study. Using a MalE-EnvZ kinase, but not the cognate partner kinase of MtrB, Rajagopalan *et al *suggested that the phosphorylation of MtrA had distinct regulation capacities. However, only 5% of the MtrA protein was shown to be phosphorylated [13]. In the present study, efforts to phosphorylate MtrA using the partner kinase of MtrB failed (data not shown). Importantly, using several different methods, we showed that the nonphosphorylated MtrA could bind to the target DNA very well, suggesting that the form of MtrA might be involved in regulating the expression of its target genes. Obviously, the regulation mechanism of MtrAB needs to be further addressed in the future.

Attempts to disrupt the *mtrA *gene in *M. tuberculosis *have been unsuccessful; thus, *mtrA *seems to be an essential gene for *M. tuberculosis *proliferation [11]. The genes encoding the MtrAB two-component system of *C. glutamicum *were deleted successfully, and this deletion strongly influenced the cell morphology, antibiotic susceptibility, and expression of genes involved in osmoprotection [15]. In the current study, a large group of target genes for MtrA was characterized from the genomes of both *M. tuberculosis *and *M. smegmatis*, including multiple transcriptional factors such as TetR family regulators, stress gene family protein (MSMEG_3308), and the isoniazid inducible protein IniA (MSMEG_0695). Inhibition of the *mtrA *gene, therefore, resulted in corresponding expression changes in many or all of these target genes in *M. smegmatis *(Fig. 5C).

In the current study, we found the conserved motifs of the MtrAB two-component system upstream of a large list of genes that have several different functions, including cell cycle progression regulation, secreted antigen, and drug resistance. Interestingly, as shown in Additional file 6, there are 42 genes (10%) that were found in both mycobacterial species. MtrA was reported to be involved in the transcriptional regulation of *dnaA *in *M. tuberculosis*; this provides the first direct evidence of its role in cell cycle progression [12]. In *M. avium*, MtrAB could respond to general stresses and ultimately inhibit cell division [14]. A recent study found that the promoter for immunodominant secreted antigen 85B was also characterized as the targets of MtrA [13]. Therefore, our findings were consistent with these previous studies.

Among the many predicted target genes, there are drug resistance and transport-related genes (including the isoniazid inducible gene *iniB*), a probable transport integral membrane gene, and osmoprotection-related genes. The operon *iniBAC *was previously found to confer multidrug tolerance to *M. bovis *BCG through an associated pump-like activity, and was induced by isoniazid and ethambutol [19,20]. These findings suggest that the *mtrA *gene might be involved in drug resistance. In the current study, we have confirmed that MtrA could bind the *iniB *promoter region. The recombinant *M. smegmatis *strain was found to become sensitive to the anti-TB drugs, isoniazid and streptomycin, when *mtrA *gene expression was inhibited by an antisense mRNA technique (Fig. 5A). In *M. avium*, *mtrAB *was shown to play a role in regulating the composition and permeability of mycobacterial cell walls and was required for morphotypic multidrug resistance [14]. In the current study, the recombinant *M. smegmatis *cells were observed to increase in length. This is most likely due to the changes of the mycobacterial cell wall, which would contribute to mycobacterial sensitivity to anti-TB drugs. All evidence makes MtrA a good target candidate for drug design.

## Conclusions

The two-component systems of *M. tuberculosis *are apparently required for its growth and resistance in hostile host environments, in which MtrAB has been reported to regulate the expression of the *M. tuberculosis *replication initiator gene, *dnaA*. In the current study, we have identified the conserved sites for the recognition of MtrA in the *dnaA *promoter as well as approximately 420 potential target genes. Further *in vivo *studies about a related organism, *M. smegmatis*, reveal changes in both cell morphology and drug resistance when MtrA gene expression is inhibited. The data presented here significantly enhance our understanding of the regulatory mechanisms of the essential two-component MtrAB system and its role in the drug resistance of *M. smegmatis*.

## Methods

### Cloning, expression and purification of recombinant proteins

All DNA primers (Additional file 7) and oligonucleotides (Additional file 8) were synthesized by Invitrogen. *M. tuberculosis mtrA *was amplified using primers from genomic DNA. The MtrA genes were cloned into the overexpression vectors pET28a or pGEX-4T-1 to produce recombinant plasmids (Additional file 1). *E. coli *BL21(DE3) cells that were transformed with the recombinant plasmid were grown at 37°C in 1 L of LB medium containing 30 μg/mL kanamycin or 100 μg/mL ampicillin, respectively. Protein purification was carried out as described in earlier reports [21-24].

### Bacterial one-hybrid analysis

The interaction between the regulatory region of the *M. tuberculosis dnaA *gene and MtrA was assayed using the bacterial one-hybrid technique [24]. The reporter vector pBXcmT and pTRG vectors containing MtrA were generated (Additional file 1). The bacterial one-hybrid assays were carried out as described in a previous study [24]. Positive growth co-transformants were selected on the screening medium plate containing 15 mM 3-AT, 8 μg/ml streptomycin, 15 μg/ml tetracycline, 34 μg/ml chloramphenicol, and 50 μg/ml kanamycin.

### Electrophoretic Mobility Shift Assay (EMSA)

The double-stranded substrates were prepared according to a previously published procedure [21]. DNA-binding assays of *M. tuberculosis *MtrA and its mutant proteins were performed using a modified electrophoretic mobility shift assay (EMSA), as previously described [21-23] but with several changes. The reactions (10 μL) for measuring the mobility shift contained 200 fmol ^32^P-labeled DNA and various amounts of MtrA diluted in a buffer containing 20 mM Tris-HCl (pH 7.5), 100 mM NaCl, 0.5 mM MgCl_2_, 10 μg/ml sonicated salmon sperm DNA, 0.7 mM 2-mercaptoethanol and 5% glycerol. Reactions were performed and gels were exposed to a storage-phosphor screen overnight at room temperature. The images were acquired using a Typhoon scanner (GE Healthcare).

### Surface Plasmon Resonance (SPR) analysis

The interaction between the regulatory region of the *M. tuberculosis dnaA *gene was assayed using SPR. Biotin-labeled promoter DNA was immobilized onto a SA chip (BIAcore), based on a previously published procedure [24]. The purified MtrA protein was passed over the chip. DNA-protein interaction assays were performed at 25°C. Each analysis was performed in triplicate. An overlay plot was generated to illustrate the interactions.

### Scanning Electron Microscopy (SEM) observation

*M. smegmatis *cells prepared for scanning electron microscopy (SEM) observation were grown in LB for 24 hours in the presence of 20 ng·mL^-1 ^tetracycline. Cells were harvested by centrifugation. The bacterial pellets were resuspended and incubated at 4°C for 24 hours in 2.5% glutardialdehyde solution. The cells were washed twice in double distilled water and then dehydrated by 15 min treatments in 30, 50, 75, 85, 95 and 100% ethanol. The incubation in 100% ethanol was repeated to ensure complete dehydration. Samples were critical-point dried, sputter-coated with gold, and observed using a scanning electron microscope (S570; Hitachi, Tokyo, Japan). Representative images are shown.

### Quantitative real-time PCR (qRT-PCR)

For real-time PCR analysis, gene-specific primers (Additional file 9) were used and first-strand cDNAs were synthesized using SuperScript II reverse transcriptase (Invitrogen), according to the manufacturer's instructions. Each PCR reaction (10 μl) contained 10 μl of 2× SYBR Green Master Mix Reagent (Applied Biosystems), 1.0 μl of cDNA samples, and 200 nM gene-specific primers. The thermocycling conditions were 95°C for 5 min, and 40 cycles at 95°C for 30 s, 60°C for 30 s and 72°C for 30 s. Amplification specificity was assessed using melting curve analysis. Different gene expressions were normalized to the levels of 16S rRNA gene transcripts [15]. The degrees of expression change were calculated using the 2^-ΔΔCt ^method [25].

### Drug resistance assays

The antimicrobial activity of four first-line anti-tuberculosis drugs against *M. smegmatis *was determined using a modified bacterial growth time course assay. *M. smegmatis *was grown in LB at 37°C overnight. This culture was then diluted (1:100) in 5 ml of fresh LB broth containing the indicated concentration of each drug, and the culture was again incubated at 37°C with shaking at 220 rpm for two days. Samples were taken at various time points (0, 6, 12, 18, 24, 30, 36, 42, and 48 h). Optical density was measured at 600 nm (OD_600_) using a Beckman DU650 spectrophotometer. All assays were performed three times. Representative growth curves are shown.

### DNase I footprinting assays

The 84 bp (S6) and 75 bp (S7) *dnaA *promoter regions were amplified (dnaAf1 and dnaAr2 were used to amplify S6 from genomic DNA, while dnaAf3 and dnaAr4 were used to amplify S7) (Additional file 7) and cleaved by endonuclease *Eco*RI, leaving a sticky 5' end that was five nucleotides from the original end. The recessive 3' end was labeled with [α-^32^P] dATP (Furui Biotech, Beijing, China) by the Klenow fragment, and then subjected to the same binding reaction as in the electrophoretic mobility shift assay. DNase I footprinting was performed as previously described [26]. The ladders were produced using the Sanger dideoxy method and dnaAf1 and dnaAf3 primers that were end-labeled by T4 polynucleotide kinase and [γ-^32^P] ATP (Furui Biotech, Beijing, China), respectively.

### Bioinformatics assays on the distribution of the identified 7 bp motif within mycobacterial genomes

The regulatory sequences were collected from the complete genomes of *M. tuberculosis *and *M. smegmatis *and the database of intergenic regions of ORFs (from stop codon to start codon) were constructed. The exact motifs (CACGCCG or CACGAGG) were then used to search for the distribution of the identified 7 bp motifs in the *M. tuberculosis *H37Rv and the *M. smegmatis *genomes. The identified target genes are listed (Additional file 10 and Additional file 11).

## Abbreviations

3-AT: 3-amino-1, 2, 4-triazole; EMSA: electrophoretic mobility shift assay; PCR: polymerase chain reaction; qRT-PCR: Quantitative real-time PCR; SA chips: Streptavidin chip; SEM: scanning electron microscopy; SPR: surface plasmon resonance; TCRs: two-component systems

## Competing interests

The authors declare that they have no competing interests.

## Authors' contributions

YL and ZGH designed the experiments. YL and JZ performed the experiments. YL HZ and ZGH analyzed the data. ZGH contributed reagents/materials/analysis tools. ZGH and YL wrote the paper. All authors have read and approved the final manuscript.

## Supplementary Material

Additional file 1 **Plasmids and recombinant vectors used in this study**. The data present plasmids and recombinant vectors used in this study. Click here for file

Additional file 2 **SPR assays for the binding of unspecific promoter chip by MtrA**. The data present SPR assays for the binding of unspecific promoter chip by MtrA. Click here for file

Additional file 3 **Competing SPR assay with the unlabeled DNA fragments for the binding of the promoter chip by MtrA**. The data present the competing SPR assay with the unlabeled DNA fragments for the binding of the promoter chip by MtrA. Click here for file

Additional file 4 **Potential target genes for MtrA in *M. tuberculosis***. The data provided potential target genes for MtrA in M. tuberculosis. Click here for file

Additional file 5 **Potential target genes for MtrA in *M.smegmatis***. The data present Potential target genes for MtrA in *M.smegmatis*. Click here for file

Additional file 6 **Homologous target genes recognized by MtrA in *M. tuberculosis *and *M. smegmatis***. The data present homologous target genes recognized by MtrA in *M. tuberculosis *and *M. smegmatis*. Click here for file

Additional file 7 **Primers used in this study**. The data provided primers used in this study. Click here for file

Additional file 8 **Sequences of the DNA substrates used in this study**. The data provided sequences of the DNA substrates used in this study. Click here for file

Additional file 9 **Primers used for quantitative real time PCR in this study**. The data present the primers used for quantitative real time PCR in this study. Click here for file

Additional file 10 **Classification and percentage of the target genes containing the 7-bp motif recognized by MtrA in *M. smegmatis***. The data present the categories and percentage of the target genes containing the 7-bp motif recognized by MtrA in *M. smegmatis*. Click here for file

Additional file 11 **The data present the categories and percentage of the target genes containing the 7-bp motif recognized by MtrA in *M. tuberculosis***. The data present the categories and percentage of the target genes containing the 7-bp motif recognized by MtrA in *M. tuberculosis*. Click here for file

## References

[B1] Johnson R Streicher EM Louw GE Warren RM van Helden PD Victor TC Drug resistance in *Mycobacterium tuberculosis* Curr Issues Mol Biol 2006 8 2 97 111 16878362

[B2] Wright A Zignol M Van Deun A Falzon D Gerdes SR Feldman K Hoffner S Drobniewski F Barrera L van Soolingen D Boulabhal F Paramasivan CN Kam KM Mitarai S Nunn P Raviglione M Global Project on Anti-Tuberculosis Drug Resistance Surveillance Epidemiology of antituberculosis drug resistance 2002-07: an updated analysis of the Global Project on Anti-Tuberculosis Drug Resistance Surveillance Lancet 2009 373 9678 1861 1873 10.1016/S0140-6736(09)60331-7 19375159

[B3] Beier D Gross R Regulation of bacterial virulence by two-component systems Curr Opin Microbiol 2006 9 2 143 152 10.1016/j.mib.2006.01.005 16481212

[B4] Stock AM Robinson VL Goudreau PN Two-component signal transduction Annu Rev Biochem 2000 69 183 215 10.1146/annurev.biochem.69.1.183 10966457

[B5] Cole ST Brosch R Parkhill J Garnier T Churcher C Harris D Gordon SV Eiglmeier K Gas S Barry CE Tekaia F Badcock K Basham D Brown D Chillingworth T Connor R Davies R Devlin K Feltwell T Gentles S Hamlin N Holroyd S Hornsby T Jagels K Krogh A McLean J Moule S Murphy L Oliver K Osborne J Deciphering the biology of *Mycobacterium tuberculosis *from the complete genome sequence Nature 1998 393 6685 537 544 10.1038/31159 9634230

[B6] Zahrt TC Deretic V *Mycobacterium tuberculosis *signal transduction system required for persistent infections Proc Natl Acad Sci USA 2001 98 22 12706 12711 10.1073/pnas.221272198 11675502 PMC60118

[B7] Ewann F Jackson M Pethe K Cooper A Mielcarek N Ensergueix D Gicquel B Locht C Supply P Transient requirement of the PrrA-PrrB two-component system for early intracellular multiplication of *Mycobacterium tuberculosis* Infect Immun 2002 70 5 2256 2263 10.1128/IAI.70.5.2256-2263.2002 11953357 PMC127906

[B8] Park HD Guinn KM Harrell MI Liao R Voskuil MI Tompa M Schoolnik GK Sherman DR Rv3133c/dosR is a transcription factor that mediates the hypoxic response of *Mycobacterium tuberculosis* Mol Microbiol 2003 48 3 833 843 10.1046/j.1365-2958.2003.03474.x 12694625 PMC1992516

[B9] Parish T Smith DA Kendall S Casali N Bancroft GJ Stoker NG Deletion of two-component regulatory systems increases the virulence of *Mycobacterium tuberculosis* Infect Immun 2003 71 3 1134 1140 10.1128/IAI.71.3.1134-1140.2003 12595424 PMC148821

[B10] Via LE Curcic R Mudd MH Dhandayuthapani S Ulmer RJ Deretic V Elements of signal transduction in *Mycobacterium tuberculosis*: in vitro phosphorylation and in vivo expression of the response regulator MtrA J Bacteriol 1996 178 11 3314 3321 8655513 10.1128/jb.178.11.3314-3321.1996 PMC178085

[B11] Zahrt TC Deretic V An essential two-component signal transduction system in *Mycobacterium tuberculosis* J Bacteriol 2000 182 13 3832 3838 10.1128/JB.182.13.3832-3838.2000 10851001 PMC94557

[B12] Fol M Chauhan A Nair NK Maloney E Moomey M Jagannath C Madiraju MV Rajagopalan M Modulation of *Mycobacterium tuberculosis *proliferation by MtrA, an essential two-component response regulator Mol Microbiol 2006 60 3 643 657 10.1111/j.1365-2958.2006.05137.x 16629667

[B13] Rajagopalan M Dziedzic R Al Zayer M Stankowska D Ouimet MC Bastedo DP Marczynski GT Madiraju MV The *Mycobacterium tuberculosis *origin of replication and the promoter for immunodominant secreted antigen 85B are the targets of MtrA, the essential response regulator J Biol Chem 2010 285 21 15816 15827 10.1074/jbc.M109.040097 20223818 PMC2871449

[B14] Cangelosi GA Do JS Freeman R Bennett JG Semret M Behr MA The two-component regulatory system mtrAB is required for morphotypic multidrug resistance in *Mycobacterium avium* Antimicrob Agents Chemother 2006 50 2 461 468 10.1128/AAC.50.2.461-468.2006 16436697 PMC1366905

[B15] Möker N Brocker M Schaffer S Krämer R Morbach S Bott M Deletion of the genes encoding the MtrA-MtrB two-component system of *Corynebacterium glutamicum *has a strong influence on cell morphology, antibiotics susceptibility and expression of genes involved in osmoprotection Mol Microbiol 2004 54 2 420 438 10.1111/j.1365-2958.2004.04249.x 15469514

[B16] Crooks GE Hon G Chandonia JM Brenner SE WebLogo: A sequence logo generator Genome Res 2004 14 6 1188 1190 10.1101/gr.849004 15173120 PMC419797

[B17] Blokpoel MC Murphy HN O'Toole R Wiles S Runn ES Stewart GR Young DB Robertson BD Tetracycline-inducible gene regulation in mycobacteria Nucleic Acids Res 2005 33 2 e22 10.1093/nar/gni023 15687380 PMC548381

[B18] Salazar L Guerrero E Casart Y Turcios L Bartoli F Transcription analysis of the *dnaA *gene and *oriC *region of the chromosome of *Mycobacterium smegmatis *and *Mycobacterium bovis *BCG, and its regulation by the DnaA protein Microbiology 2003 149 Pt 3 773 784 10.1099/mic.0.25832-0 12634345

[B19] Colangeli R Helb D Sridharan S Sun J Varma-Basil M Hazbón MH Harbacheuski R Megjugorac NJ Jacobs WR Jr Holzenburg A Sacchettini JC Alland D The *Mycobacterium tuberculosis *iniA gene is essential for activity of an efflux pump that confers drug tolerance to both isoniazid and ethambutol Mol Microbiol 2005 55 6 1829 1840 10.1111/j.1365-2958.2005.04510.x 15752203

[B20] Alland D Steyn AJ Weisbrod T Aldrich K Jacobs WR Jr Characterization of the *Mycobacterium tuberculosis *iniBAC promoter, a promoter that responds to cell wall biosynthesis inhibition J Bacteriol 2000 182 7 1802 1811 10.1128/JB.182.7.1802-1811.2000 10714983 PMC101861

[B21] He ZG Rezende LF Willcox S Griffith JD Richardson CC The carboxyl-terminal domain of bacteriophage T7 single-stranded DNA-binding protein modulates DNA binding and interaction with T7 DNA polymerase J Biol Chem 2003 278 32 29538 29545 10.1074/jbc.M304318200 12766155

[B22] Jiang PX Wang J Feng Y He ZG Divergent functions of multiple eukaryote-like Orc1/Cdc6 proteins on modulating the loading of the MCM helicase onto the origins of the hyperthermophilic archaeon *Sulfolobus solfataricus *P2 Biochem Biophys Res Commun 2007 361 3 651 658 10.1016/j.bbrc.2007.07.073 17673179

[B23] Wang J Jiang PX Feng H Feng Y He ZG Three eukaryote-like Orc1/Cdc6 proteins functionally interact and mutually regulate their activities of binding to the replication origin in the hyperthermophilic archaeon *Sulfolobus solfataricus *P2 Biochem Biophys Res Commun 2007 363 1 63 70 10.1016/j.bbrc.2007.08.125 17825793

[B24] Guo M Feng H Zhang J Wang W Wang Y Li Y Gao C Chen H Feng Y He ZG Dissecting transcription regulatory pathways through a new bacterial one-hybrid reporter system Genome Res 2009 19 7 1301 1308 10.1101/gr.086595.108 19228590 PMC2704442

[B25] Livak KJ Schmittgen TD Analysis of relative gene expression data using real-time quantitative PCR and the 2^-ΔΔCt ^method Methods 2001 25 4 402 408 10.1006/meth.2001.1262 11846609

[B26] Yin P Li TY Xie MH Jiang L Zhang Y A type Ib ParB protein involved in plasmid partitioning in a Gram-positive bacterium J Bacteriol 2006 188 23 8103 8108 10.1128/JB.01232-06 16997970 PMC1698188

