# Supplementary material for: The characterization of conserved binding motifs and potential target genes for M. tuberculosis MtrAB reveals a link between the two-component system and the drug resistance of M. smegmatis
Source: BMC Microbiol. 2010 Sep 16;10:242. doi: 10.1186/1471-2180-10-242 (PMC2945938; doi:10.1186/1471-2180-10-242)
Supplement: Additional file 1 — Plasmids and recombinant vectors used in this study. The data present plasmids and recombinant vectors used in this study. [file 1471-2180-10-242-S1.DOC]

**Additional file 1.** Plasmids and recombinant vectors used in this study

| **Plasmids** | **Description** | **Sources** |
| --- | --- | --- |
| pBT | Bacterial two-hybird assay bait domain vector | Stratagene |
| pTRG | Bacterial two-hybird assay target domain vector | Stratagene |
| pTRG-MtrA | pTRG derivative for Bacterial one-hybird assay | This work |
| pET28a | Kan^r^ expression vector with 6His-tag coding sequence | Novagen |
| pET-MtrA | pET derivative for expression 6His-MtrA | This work |
| pBXcmT | pBT derived plasmid for detection of protein-DNA interaction | *Guo et al*, 2009 |
| pBX-*dnaAp* | pBXcmT derivative for Bacterial one-hybird assay | This work |
| pBX-SsoDNA | pBXcmT derivative for Bacterial one-hybird assay, containing an archaeal DNA fragment | This work |
| pMind | A tetracycline-inducible system for conditional gene silencing in mycobacteria | *Blokpoel et al*, 2005 |
| pMind-MtrAantisense | pMind derivative for silencing of *mtrA* in *M.sm* | This work |
