# Supplementary material for: The characterization of conserved binding motifs and potential target genes for M. tuberculosis MtrAB reveals a link between the two-component system and the drug resistance of M. smegmatis
Source: BMC Microbiol. 2010 Sep 16;10:242. doi: 10.1186/1471-2180-10-242 (PMC2945938; doi:10.1186/1471-2180-10-242)
Supplement: Additional file 2 — SPR assays for the binding of unspecific promoter chip by MtrA. The data present SPR assays for the binding of unspecific promoter chip by MtrA. [file 1471-2180-10-242-S2.DOC]

**Additional file 2**


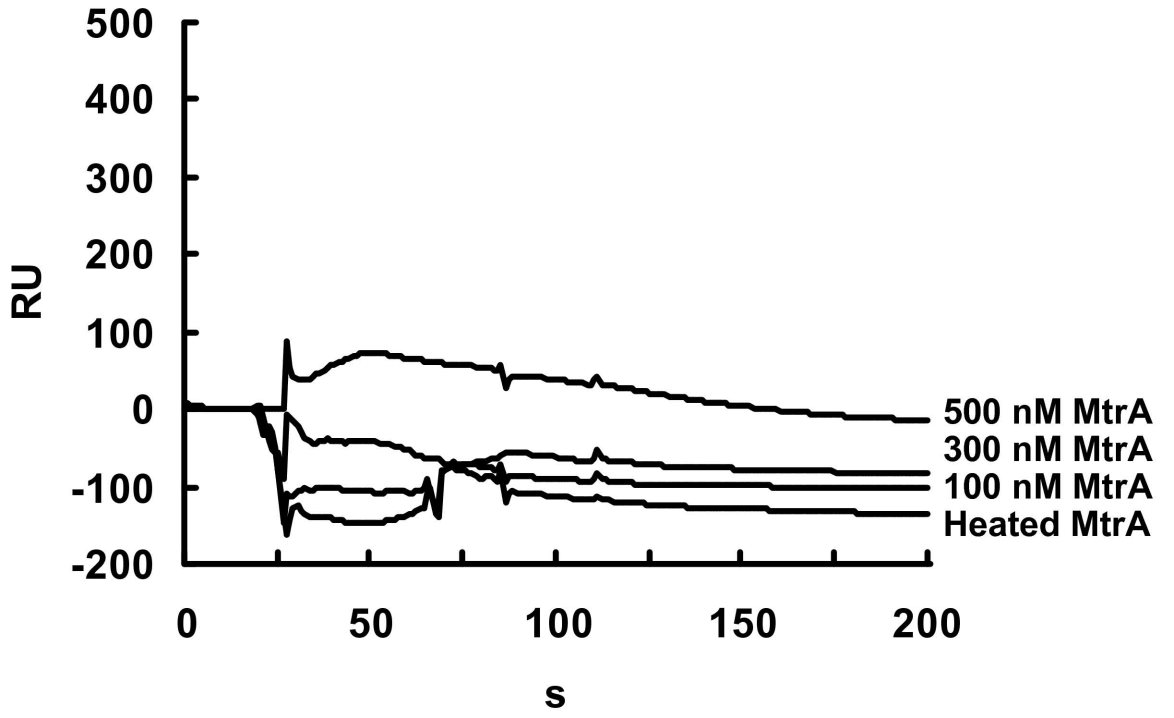


**SPR assays for the binding of unspecific promoter chip by MtrA**. The DNA-binding assays of *M. tuberculosis* MtrA were performed using SPR assays as described in the “Materials and Methods”. An unspecific DNA, the promoter of Rv0467, was coated on the chip, the purified MtrA protein was passed over the chip.
