# Supplementary material for: The characterization of conserved binding motifs and potential target genes for M. tuberculosis MtrAB reveals a link between the two-component system and the drug resistance of M. smegmatis
Source: BMC Microbiol. 2010 Sep 16;10:242. doi: 10.1186/1471-2180-10-242 (PMC2945938; doi:10.1186/1471-2180-10-242)
Supplement: Additional file 3 — Competing SPR assay with the unlabeled DNA fragments for the binding of the promoter chip by MtrA. The data present the competing SPR assay with the unlabeled DNA fragments for the binding of the promoter chip by MtrA. [file 1471-2180-10-242-S3.DOC]

**Additional file 3**


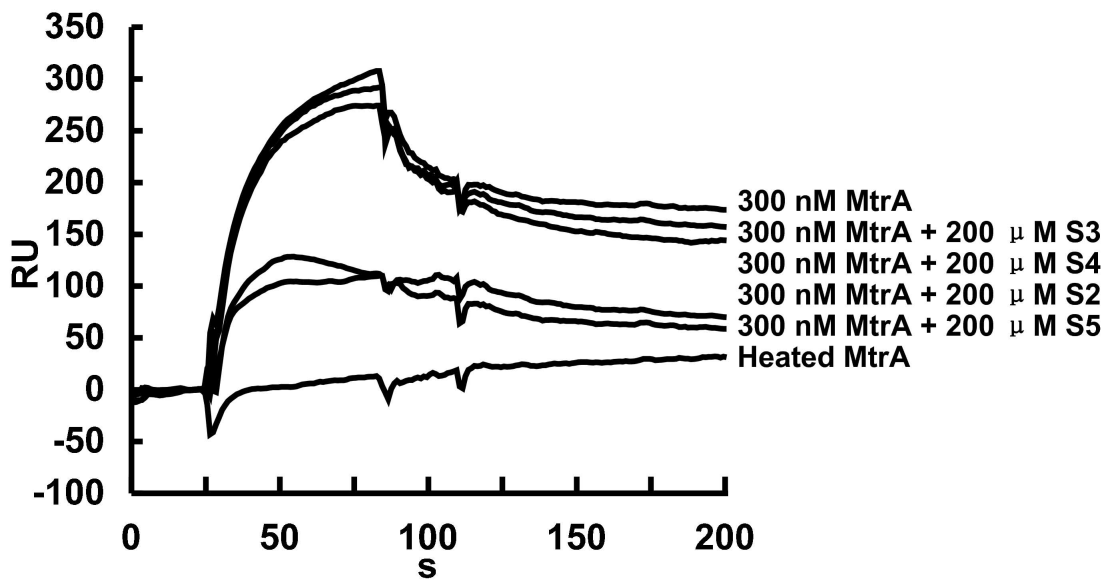


**Competing SPR assay with the unlabeled DNA fragments for the binding of the promoter chip by MtrA**. The DNA-binding assays of *M. tuberculosis* MtrA were performed using SPR assays as described in the “Materials and Methods”. Significantly lower responses were observed for S2 and S5, both of which contain MtrA binding motif, indicating that they could compete the binding of MtrA to the promoter on the chip.
