# Supplementary material for: The characterization of conserved binding motifs and potential target genes for M. tuberculosis MtrAB reveals a link between the two-component system and the drug resistance of M. smegmatis
Source: BMC Microbiol. 2010 Sep 16;10:242. doi: 10.1186/1471-2180-10-242 (PMC2945938; doi:10.1186/1471-2180-10-242)
Supplement: Additional file 7 — Primers used in this study. The data provided primers used in this study. [file 1471-2180-10-242-S7.DOC]

**Additional file 7**. Primers used in this study.

| **Construct** | **Primer name** | **Sequence (from 5' to 3')** |
| --- | --- | --- |
| MtrA | *R3246-f*  *R3246-r* | GCGCGCGGCCGCGATGGACACCATGAGGCAAAGGA  CCCGTCTAGATCACGGAGGTCCGGCCTTGTACCCC |
| MtrAantisense | *MtrAantisense-f*  *MtrAantisense-r* | CGCGCGACTAGTATGGACACCATGAGGCAAAG  TATATACTGCAGTCACGGGGGTCCGGCCTTGT |
| BIO-dnaAp | *Dnapf*  *Dnapr* | Biotin-TAATCCAGGTCGCAGCCGCATCG  CGACGTATCTCCCTGGTTCTCGT |
| DNA substrate S6 | *dnaAf1*  *dnaAr2* | TACGGAATTCTACGGAAAACTGTTAGCTTC  GCCATCCTCGCTTGATTGTC |
| DNA substrate S7 | *dnaAf3*  *dnaAr4* | TACGGAATTCTAGCGGCCCGGACAACTTGA  ACCAGCCGCAACCTACCGGC |
