# Supplementary material for: The characterization of conserved binding motifs and potential target genes for M. tuberculosis MtrAB reveals a link between the two-component system and the drug resistance of M. smegmatis
Source: BMC Microbiol. 2010 Sep 16;10:242. doi: 10.1186/1471-2180-10-242 (PMC2945938; doi:10.1186/1471-2180-10-242)
Supplement: Additional file 8 — Sequences of the DNA substrates used in this study. The data provided sequences of the DNA substrates used in this study. [file 1471-2180-10-242-S8.DOC]

**Additional file 8**. Sequences of the DNA substrates used in this study.

| **Name** | **Sequence (from 5' to 3')** |
| --- | --- |
| *S1* | GCGGTGTAGTTATCACGCCG |
| *S2* | TCACGCCGTTTCAGCGTGGAAACGGCACTC |
| *S3* | TTTCAGCGTGGAAACGGCACTC |
| *S4* | GCGAGCCGTTGCCGGTAGGTTGCGGCTGGT |
| *S5* | AACACGAGGATCGCGAGCCGTTGCCGGTAGGTTGCGGCTGGT |
| *Negative control* | CGGAGGTGGTTTTCGCTGCT |
| *MtbdnaA1p* | TAGTTATCACGCCGTTTCAGC |
| *Rv0341p* | CGCCGGTCACGCCGGCGAACA |
| *Rv0574cp* | GCGGTCTTGAGGACCTTCGGCCCCACCCACGAGGCCGCCG |
| *Rv3476cp* | CTGCATCCACGCCGGCGACACGCCGAGGGACG |
| *CgluproPp* | GACTTTTCACCCCGAACCTTA |
| *CglumepAp* | CCCCTAGCACGGCGTGTTTGG |
