# Supplementary material for: The characterization of conserved binding motifs and potential target genes for M. tuberculosis MtrAB reveals a link between the two-component system and the drug resistance of M. smegmatis
Source: BMC Microbiol. 2010 Sep 16;10:242. doi: 10.1186/1471-2180-10-242 (PMC2945938; doi:10.1186/1471-2180-10-242)
Supplement: Additional file 9 — Primers used for quantitative real time PCR in this study. The data present the primers used for quantitative real time PCR in this study. [file 1471-2180-10-242-S9.DOC]

**Additional file 9. Primers used for quantitative real time PCR in this study**

| Primer name | Sequence (from 5' to 3') |
| --- | --- |
| MSMEG_0321F | CGGCTCTCAGCTGTTCATCA |
| MSMEG_0321R | CCAGCTTCACGAACACCTTTC |
| MSMEG_0343F | CCGCCACTTCGAGAGCAA |
| MSMEG_0343R | TCGTCTATCACCTGGTCGAACA |
| MSMEG_0695F | AAGTTCCTCTCCGAGCATGTG |
| MSMEG_0695R | CGGCCAGCAGCAGATGTT |
| MSMEG_0983F | TCACGGCATCGGCTATCTG |
| MSMEG_0983R | CTCGAGGGTCTCGACGAAGT |
| MSMEG_0985F | GGCTACGTGACCGTCTGCAT |
| MSMEG_0985R | CCAGGTAGGTCTCGGACTTGTT |
| MSMEG_1398F | CAGGTTGAGGTGACCGCTTAC |
| MSMEG_1398R | CACCATCGAGTGCTCCTGAA |
| MSMEG_1556F | GCATGCTTCCGCACAACA |
| MSMEG_1556R | GGGACCCGCGTACACCTT |
| MSMEG_1831F | AGAGGCCAAGCGCATCTG |
| MSMEG_1831R | CAGATACCGAAGCGCTCATCA |
| MSMEG_1874F | CGTTTGGTGAACGTGCATGT |
| MSMEG_1874R | GGACCACCTGCGGGTTCT |
| MSMEG_1875F | TGGCCGAAAGCCTGTCA |
| MSMEG_1875R | TGGCTCACGTCAGAGGTGAA |
| MSMEG_1883F | CTAAATTTGACGCGGACGAAA |
| MSMEG_1883R | CGCTACCGACGGGTGATT |
| MSMEG_2557F | TCTCCAGACCCACGCTGTAC |
| MSMEG_2557R | CGTTCCCACACCACGAA |
| MSMEG_3275F | GTCGTGTTCGAGGTGCTGAA |
| MSMEG_3275R | GCAAGGGCACGGTTGTG |
| MSMEG_3288F | ATCGCACGGCAGAACCA |
| MSMEG_3288R | TGACGTAGGGAACGAGGATCTC |
| MSMEG_3308F | AGAACGCGAAGCTCATC |
| MSMEG_3308R | CCCATCCACCCTTCTC |
| MSMEG_3321F | CGCATCACTCGGAGTTGGT |
| MSMEG_3321R | CGGCCTTGGTTGGTGATC |
| MSMEG_3332F | GCACATTGTTCGGATTCCTCTAC |
| MSMEG_3332R | CATCACGTGGTCGGTTTCCT |
| MSMEG_3354F | CCTCAGTGACGCAGCGAAAT |
| MSMEG_3354R | CCAGAATCTCCTCGGCGATA |
| MSMEG_3572F | ATCGTCCGGTTGTTCGA |
| MSMEG_3572R | CACCACGTTGCGGTACAT |
| MSMEG_3757F | AGCAGCCGCGGTAATACG |
| MSMEG_3757R | CGAGCTCTTTACGCCCAGTAA |
| MSMEG_4213F | CGACGAGATCTTCAAGATGTTCA |
| MSMEG_4213R | ACGCGTCGAGTTCTTTCCA |
| MSMEG_4236F | GTCACCAAGAGCCAAGATCACA |
| MSMEG_4236R | GGGCCAGTTTCTCGAACTCA |
| MSMEG_4256F | GGGCGACCTGGTGACCTAT |
| MSMEG_4256R | TCATTCCGTCTCCGATGTAGATAC |
| MSMEG_4614F | CACCTTGAGATCGTGGTTATCG |
| MSMEG_4614R | CTTCAGATTCCTCAAGCCACTACA |
| MSMEG_5338F | GGGTCCGACTCTCACCAGAA |
| MSMEG_5338R | TCGGCAGTCCGGTAGATGAT |
| MSMEG_5439F | GGCGACCAGAGAAGAACAAATT |
| MSMEG_5439R | GCCAGGCTCCCCAACCT |
| MSMEG_5853F | CAGGGCGAGTCGATATTCGA |
| MSMEG_5853R | ACGCGAGATGGTCTTGATGTC |
| MSMEG_6897F | TCCCTGGAGACGTTCCTGAA |
| MSMEG_6897R | GCCCCAGATGTCAACCTTGT |
| [MSMEG_6947F](http://www.ncbi.nlm.nih.gov/entrez/utils/fref.fcgi?http://www.tigr.org/tigr-scripts/CMR2/GenePage.spl?locus=MSMEG_6947) | CCGGGTATGCGTGTCAAGTA |
| [MSMEG_6947R](http://www.ncbi.nlm.nih.gov/entrez/utils/fref.fcgi?http://www.tigr.org/tigr-scripts/CMR2/GenePage.spl?locus=MSMEG_6947) | TCACGCAGCGAGTTGATGA |
