# Supplementary material for: The characterization of conserved binding motifs and potential target genes for M. tuberculosis MtrAB reveals a link between the two-component system and the drug resistance of M. smegmatis
Source: BMC Microbiol. 2010 Sep 16;10:242. doi: 10.1186/1471-2180-10-242 (PMC2945938; doi:10.1186/1471-2180-10-242)
Supplement: Additional file 11 — The data present the categories and percentage of the target genes containing the 7-bp motif recognized by MtrA in M. tuberculosis. The data present the categories and percentage of the target genes containing the 7-bp motif recognized by MtrA in M. tuberculosis. [file 1471-2180-10-242-S11.DOC]

**Additional file 11**


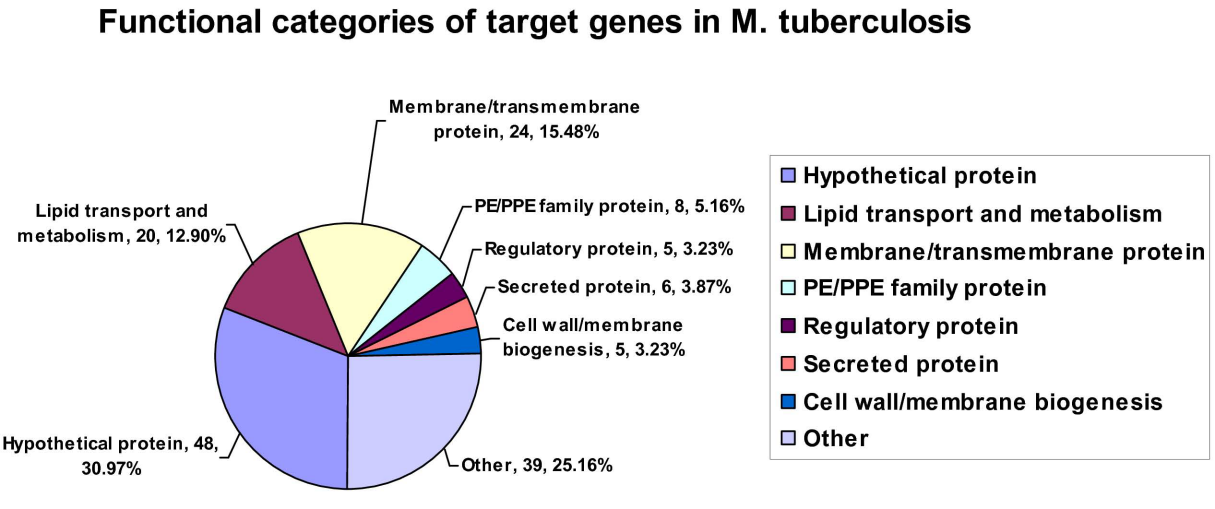


**Classification and percentage of the target genes containing the 7-bp motif recognized by MtrA in *M. tuberculosis*.**
